# The Fabrication of Solid Polymer Electrolyte from CS/PEO/NaClO_4_/Fly Ash Composite

**DOI:** 10.3390/polym14224792

**Published:** 2022-11-08

**Authors:** Yatim Lailun Ni’mah, Mohamat Ashar Eka Saputra, Suprapto Suprapto, Hamzah Fansuri, Putu Suwarta, Achmad Subhan, Sylvia Ayu Pradanawati

**Affiliations:** 1 Department of Chemistry, Faculty of Science and Data Analytics, Institut Teknologi Sepuluh Nopember, Surabaya 60111, Indonesia; 2 Mechanical Engineering Department, Institut Teknologi Sepuluh Nopember, Surabaya 60111, Indonesia; 3 Research Center for Advanced Materials-National Research and Innovation Agency, Tangerang Selatan 15314, Indonesia; 4 Mechanical Engineering Department, Universitas Pertamina, Daerah Khusus Ibukota Jakarta 12220, Indonesia

**Keywords:** chitosan, fly ash, PEO, ionic conductivity, sodium-ion battery

## Abstract

Solid polymer electrolytes (SPEs) have been successfully fabricated from CS/PEO/NaClO_4_/Fly ash composite. Chitosan (CS), an organic polymer, was blended with polyethylene oxide (PEO) to enhance its electrochemical properties. However, SPEs based on CS/PEO composites have low conductivity. Fly ash (FA) has been studied to be used as a filler to increase the ionic conductivity of SPEs. In this study, polymer composites based on CS and PEO were developed with the addition of FA as a filler using the solution casting method. The interactions between CS, PEO, NaClO_4_, and fly ash were observed using FTIR. The SPE characterization using XRD and DSC showed a decrease in crystallinity after the addition of NaClO_4_ and FA. The SPE composite morphology and elemental distribution were investigated using SEM. SPE conductivity analysis using EIS showed the optimum results for SPE fabricated with a ratio of CS:PEO:NaClO_4_ = 3:2:7.5, which was 1.02 × 10^−4^ S cm^−1^ at 30 °C and increased to 2.13 × 10^−3^ S cm^−1^ at 60 °C. The addition of FA (5 wt.%) increased the conductivity to 3.20 × 10^−4^ S cm^−1^ at 30 °C and increased to 4.34 × 10^−3^ S cm^−1^ at 60 °C.

## 1. Introduction

A battery is an energy storage device that is widely used in everyday portable electronic devices, such as watches, children’s toys, gadgets, and laptops. The most common battery used in portable devices is the lithium-ion battery (LIB). However, lithium as a raw material for LIBs has a limited abundance in nature (2.00 × 10^−3^ *w*/*w* in the earth’s crust), which may limit its implementation in the future [1]. The sodium-ion battery (SIB) is an alternative to LIBs because of the abundance of sodium as a raw material for SIBs in nature. The natural reserve of sodium is higher than lithium (Li:Na = 1:1000). On the other hand, SIBs and LIBs have a comparable performance [2]. Sodium has a suitable redox potential to replace lithium (E^0^ Na^+^/Na = −2.71 V vs. SHE, while Li^+^/Li = −3.045 V vs. SHE). The sodium reduction potential is only 0.30 V above lithium [3].

The battery material’s composition and configuration affect the battery’s performance. In general, the electrolytes in the battery are liquid. Liquid electrolyte causes several problems in their application, such as leakage, flammability, and a rapid decrease in battery performance [1,4]. Solid polymer electrolytes (SPEs) can be used as an alternative to liquid electrolytes as they are lightweight, have high flexibility, and have no leakage risk [5,6,7]. However, SPEs have low ionic conductivity at room temperature. The low ionic conductivity limits the future application of SPEs, so their conductivity needs to be increased.

CS is a biopolymer that can be used in electrochemical devices, such as electronic devices, sensors, batteries, and fuel cells [8,9]. CS is used as a host polymer for SPE materials because it forms a good film and it is inexpensive, compatible with various organic acid solvents, environmentally friendly, and easy to manufacture [5]. Furthermore, CS has the second-highest natural abundance after cellulose. CS was found in the exoskeleton of crustaceans, fungal cell walls, and other biological materials [10,11]. However, the rigid structure of the CS chain makes the CS film brittle, and it has poor physical properties [12]. CS has a low ionic conductivity at room temperature (10^−8^ S cm^−1^) [13]. Several approaches have been investigated to improve the physical properties and ionic conductivity of SPEs, i.e., blending with other polymers, adding plasticizers, adding fillers, and grafting [14].

PEO is a polymer that is widely used for SPEs. This is because PEO has higher electrochemical stability than other polymer electrolytes [1]. PEO is a semi-crystalline polymer, which leads to low conductivity values at high temperatures (10^−6^–10^−8^ S cm^−1^) [15,16]. Blending CS with PEO can improve the thermal properties and ionic conductivity of SPEs due to the interaction between amino groups (proton donors) and ether groups (proton acceptors) in the polymer blend [17,18,19]. Amorphous CS chains can suppress PEO crystallinity and the rigidity of the CS chain can be complemented by flexible PEO chains [12]. In addition, the salt concentration also affects the ionic conductivity of the SPE. The higher the salt concentration, the lower the degree of dissociation [20]. This becomes the basis for optimizing the salt concentration to improve ionic conductivity. At the optimum salt concentration, the maximum number of free ions was formed. 

In the host polymer, the ion transport is caused by the segmental movement of the polymer. Hence, the ion movement is faster in the amorphous phase than in the crystalline phase of the host polymer [21]. The addition of filler can suppress the crystallinity of the SPE host polymer, allowing ions to move more easily so that conductivity increases. Al_2_O_3_, SiO_2_, TiO_2,_ and Na_3_N have been studied to be applied as fillers [1,22,23]. Fly ash is a coal-burning product in power plants. Fly ash contains many metal oxides in the form of SiO_2_, Al_2_O_3_, and Fe_2_O_3_ [24].

Based on the presented background, in this study, fly ash was used as a filler which was added to a mixture of PEO and a chitosan polymer with NaClO_4_ was used as an electrolyte salt. The metal oxides in the fly ash were used as fillers. In addition, the NaClO_4_ salt content in SPE production was optimized. Therefore, the ionic conductivity value of the SPE was expected to increase.

## 2. Materials and Methods

### 2.1. Materials

Materials used in this research included polyethylene oxide (PEO, MW 600,000 g mol^−1^, Sigma-Aldrich, Taufkirchen, Germany), sodium perchlorate (NaClO_4_, Merck, Darmstadt, Germany, ≥98.0%), glacial acetic acid (Sigma-Aldrich, Anhui, China, CAS No. 64-19-7), and chitosan crab (Pharma grade, Chimultiguna, Bogor, Indonesia) with DD 97% and fly ash obtained from PT Paiton, Probolinggo, East Java, Indonesia.

### 2.2. Preparation and Salt Optimization of Solid Polymer Electrolyte

SPE was synthesized by mixing CS, PEO, and NaClO_4_. CS and PEO in PTFE bottles at a ratio of 3:2, as well as acetic acid 1%, were added [25]. The mixture was stirred at room temperature for 6 h until a homogeneous solution formed. NaClO_4_ salt was added to the solution with different ratios of CS, PEO, and NaClO_4_, namely CS:PEO:NaClO_4_ = 3:2:0; 3:2:4.5; 3:2:6; 3:2:7.5; 3:2:9, as tabulated in Table 1. The mixture was stirred further for 1 h until a homogeneous solution was formed [26]. The homogeneous mixture was poured into a 2 cm diameter Teflon mold, and the solvent was evaporated at room temperature for 48 h.

### 2.3. Preparation of Composite Solid Polymer Electrolyte with Fly Ash as Filler

CS and PEO were mixed in a Teflon bottle, and acetic acid 1% was added dropwise. The mixture was stirred at room temperature for 6 h until a homogeneous solution was obtained. NaClO_4_, with a ratio of CS:PEO: NaClO_4_ = 3:2:7.5, and fly ash, with a concentration of 5% *w*/*w*, were added to the mixture. The mixture was stirred for 1 h until a homogeneous solution was obtained. The mixture was then poured into a 2 cm diameter Teflon mold and the solvent was evaporated at room temperature for 48 h.

### 2.4. Characterization

X-ray diffraction (XRD) characterization was performed using a Philips X-Pert (Worcestershire, UK) with a Cu-Kα radiation source (λ = 1.5405, 40 kV, and 30 mA). Samples were analyzed at 2θ = 5°–60° with a scan rate of 0.5 °/min at room temperature.

The FTIR analysis was performed using Agilent Cary 630 FTIR Spectrometer (Santa Clara, USA) with a wavelength of 4000–400 cm^−1^ at room temperature. Thermal analysis of the SPE was performed using a differential scanning calorimetry (DSC) Perkin Elmer Jade (Shelton, CT, USA) on a gold-plated high-pressure stainless-steel capsule. The sample was put in a capsule and placed in a glove box under an argon atmosphere. The DSC measurement was carried out at temperatures of 25 °C to 300 °C with a heating rate of 10 °C/min [27].

A scanning electron microscopy (SEM, Zeis EVO MA 10, Jena, Germany) analysis was performed to observe the surface morphology of the SPE. The SEM examination was performed under a vacuum and the surface of the sample was coated with gold–palladium. The ionic conductivity of the SPE film was measured using electrochemical impedance spectroscopy (EIS). The test was performed by placing a foil between two stainless steels. The EIS analysis was performed with Metrohm AutoLab PGSTAT302N (Utrecht, Netherland) with a frequency range of 1 MHz–1 Hz and a voltage of 10 mV. The ionic conductivity of the sample was calculated using Equation (1) [28]:

(1) $$\sigma =\frac{t}{RA}$$

where σ is the conductivity, *t* is the film thickness, *R* is the resistivity, and *A* is the effective film–electrode contact area. The relationship between ionic conductivity and temperature was measured from 30–90 °C.

## 3. Results

### 3.1. Preparation of Solid Polymer Electrolyte and Salt Optimization

**Figure 1:**
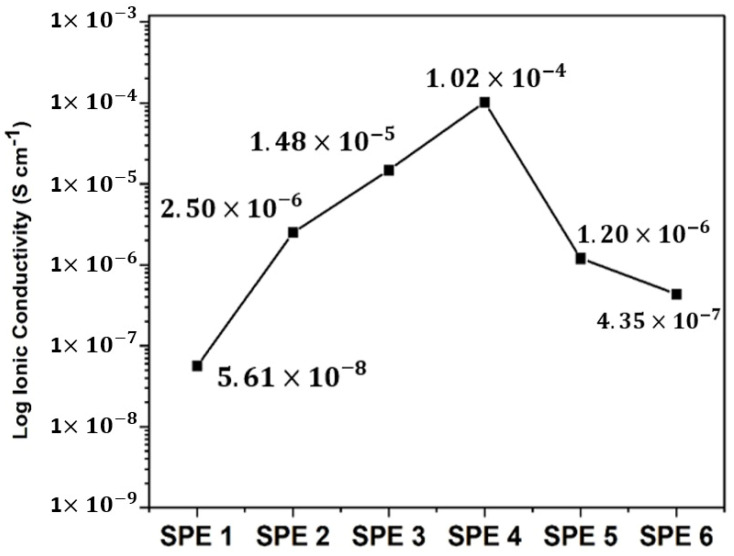
Ionic conductivity at various ratios of NaClO_4_.

The ionic conductivity from the optimization of the NaClO_4_ salt concentration is shown in Figure 1. The ionic conductivity of the SPE increased after the addition of NaClO_4_ salt. The conductivity of the pure CS and PEO ions was the same. The conductivity of the SPE ions increased after the addition of NaClO_4_ salt. The conductivity of the pure CS/PEO SPE was 5.61 × 10^−8^ S cm^−1^ and increased to 2.50 × 10^−6^ S cm^−1^ in the sample SPE1. The addition of NaClO_4_ salt as a source of sodium ions that mobilize into SPEs increases its ionic conductivity. The CS/PEO SPE conductivity increased when the NaClO_4_ salt was added, but no increase was shown in conductivity for the CS:PEO: NaClO_4_ ratio above 3:2:7.5. Arof et. al., 2015, reported a similar finding for the CS/PEO/NH_4_NO_3_ electrolyte system [28], where the ionic conductivity increased with the increasing concentration of the ammonium salt and reached a maximum point at the concentration of NH_4_NO_3_:CS = 1. This can be attributed to the ion pairing at the salt concentration [29]. However, a higher concentration of Na^+^ ions makes the SPE difficult to mold [1].

### 3.2. Preparation of Composite Solid Polymer Electrolyte with Fly Ash as Filler

**Figure 2:**
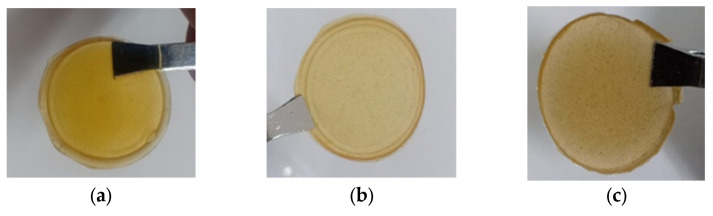
SPE, (**a**) pure CS/PEO, (**b**) CS/PEO/NaClO_4_ (CS:PEO:NaClO_4_ = 3:2:7.5), (**c**) CS/PEO/NaClO_4_/fly ash 5%.

The SPE composite film produced is shown in Figure 2. The combination of CS and PEO produced a film that was hard, less elastic, and dark yellow. The addition of NaClO_4_ caused the color of the film to become light yellow. In addition, the addition of fly ash resulted in a brown film. The addition of fly ash suppressed the crystallinity of the SPE film to facilitate the mobilization of sodium ions and ultimately increase SPE ionic conductivity. The filler added was to 5 wt% of the polymer blend film’s total mass according to studies by Ni’mah (2018) [24].

The composition of the fly ash used in this research is shown in Table 2. There were three major components in the fly ash: SiO_2_, Al_2_O_3_, and Fe_2_O_3_. Other components, such as Ti, P, Mg, Ca, Zn, and Sr oxides, were present as minor components. Metal oxides, such as Al_2_O_3_, TiO_2_, SiO_2,_ and Fe_2_O_3_, were studied as fillers in the SPE. Therefore, fly ash from PT. Paiton, Probolinggo, East Java, Indonesia can be used as an SPE filler.

**Figure 3:**
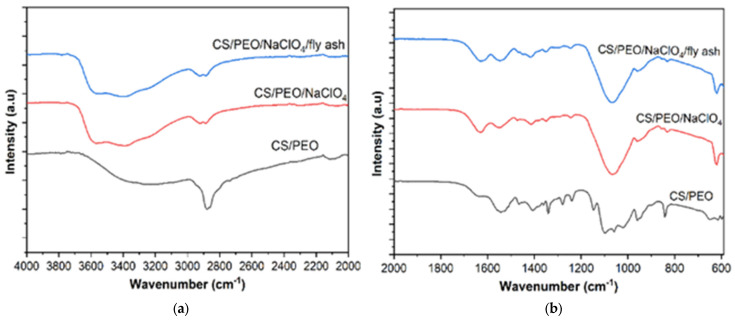
(**a**) FTIR spectra of SPE, (**b**) The SPE FTIR Spectra from 600 to 2000 cm^–1^.

The FTIR spectra of the CS/PEO, CS/PEO/NaClO_4_, and CS/PEO/NaClO_4_/fly ash SPE are shown in Figure 3. FTIR analysis of the SPE was used to determine the interactions between CS, PEO, NaClO_4_, and fly ash. The broad peak at a wavenumber of 3538 cm^−1^ was due to O-H group vibration. The vibrations of CH_2_ occurred at wavenumbers 2884, 1464, 1402, 1341, 1360, 1282, and 1237 cm^−1^. The absorption band of C=O-NHR was observed at a wavenumber of 1640 cm^−1^ and the absorption band of amine-NH_2_ was observed at 1543 cm^−1^. The stretching vibrations of the C-O-C bonds appeared at the peaks of 1144, 1095, and 1058 cm^−1^. 

The addition of NaClO_4_ decrease the intensity of C-H aliphatic vibration as shown in Figure 3a. The peak at wavenumbers of 600–650 cm^−1^ was observed after the addition of NaClO_4_ salt to CS/PEO and CS/PEO/fly ash samples. The peak was from ClO_4_^−^ and indi-cated that there was a reaction between CS/PEO and NaClO_4_ since this peak did not ap-pear in pure CS/PEO, as shown in Figure 3b. Furthermore, the SPE spectra with and without the addition of fly ash were not significantly different, indicating there were no new bonds formed.

**Figure 4:**
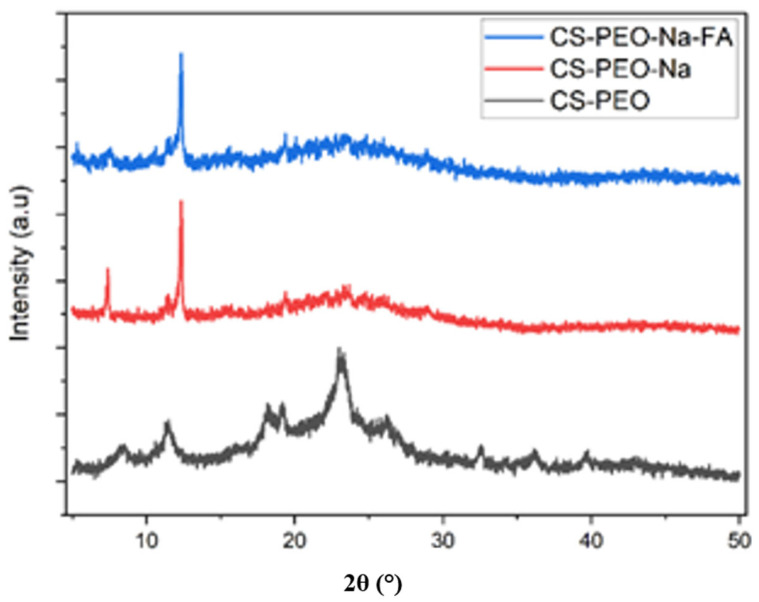
XRD diffractogram of SPE.

The XRD Diffractogram of the CS/PEO, CS/PEO/NaClO_4_, and CS/PEO/NaClO_4_/fly ash SPE are shown in Figure 4. The pure CS/PEO XRD diffractogram showed a characteristic sharp peak at 2θ = 11.42°, 18.14°, 19.18°, and 22.96° with the base peak at 2θ = 8.40°, 26.23°, 32.57°, 36.20°, and 39.68°. The characteristic sharp peak indicates the semi-crystalline region, while the base peak indicates the amorphous region. When NaClO_4_ was added, it effectively reduced the peak intensity and crystallinity of pure CS/PEO. This occurred due to the disruption of the semi-crystalline structure by the addition of NaClO_4_ salt. When 5% of the fly ash filler was added, there was a decrease in the intensity of the XRD characteristic peak, indicating a decrease in the degree of crystallinity of the CS/PEO backbone. The fly ash was homogeneously dispersed and filled the gaps between the CS and PEO chains, preventing or hindering the crystallization of the CS and PEO chains due to the large surface area [30].

**Figure 5:**
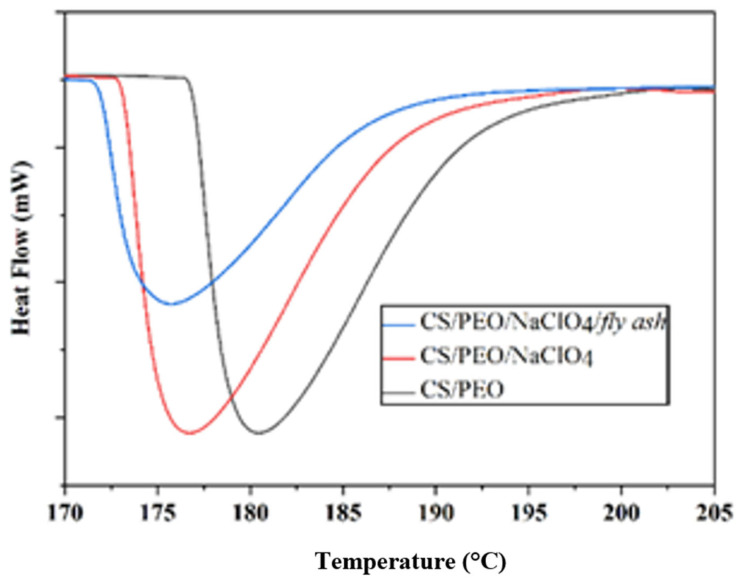
DSC curve of SPE.

The DSC thermal analysis curves of the CS/PEO, CS/PEO/NaClO_4_, and CS/PEO/NaClO_4_/fly ash SPE are shown in Figure 5. The SPE-CS/PEO samples had a melting point of 180.23 °C. The addition of NaClO_4_ salt to SPE CS/PEO caused the intensity of the endothermic peak and the melting point to decrease to 176.51 °C. The addition of fly ash lowered the melting point to 175.53 °C. The lower melting point (Tm) was due to the decreasing the SPE crystallinity degree after the addition of NaClO_4_ and fly ash, according to data obtained from the XRD.

XRD and DSC analysis showed that the addition of NaClO_4_ and fly ash to CS/PEO reduced the crystallinity and melting point (Tm), which was confirmed by the percent relative crystallinity (*Xc*), calculated using Equation (2):

(2) $$X_{C\;}=\frac{∆H_{m,PEO}}{∆H_{m,PEO}^{0}\times C}$$

where $∆H_{m,PEO}$ is melting enthalpy from the sample, $∆H_{m,PEO\;}^{0}$ is the melting enthalpy of 100% crystalline PEO (203 J/g for PEO with MW 600.000 g/mol), and *C* is the weight fraction from PEO in the polymer blend. The calculated relative crystallinity is summarized in Table 3. The crystallinity values decreased after the addition of NaClO_4_ and fly ash.

The decrease in crystallinity degree makes the SPE properties change. The amorphous polymer host facilitates the mobilization of Na^+^ ions in the matrix by increasing the mobilization of Na^+^ ions. The increase in charge mobility increases SPE conductivity [31].

**Figure 6:**
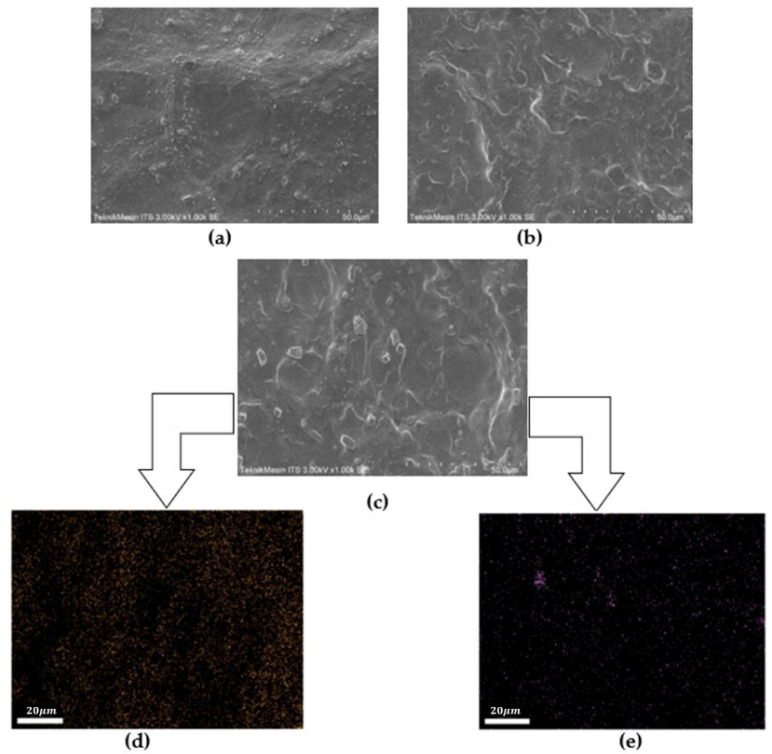
SEM micrograph of: (**a**) pure CS/PEO; (**b**) CS/PEO/NaClO_4_; (**c**) CS/PEO/NaClO_4_/fly ash; and (**d,e**) Na dan Si elemental mapping of CS/PEO/NaClO_4_/fly ash.

The SEM analysis of SPE films, CS/PEO, CS/PEO/NaClO_4_, and CS/PEO/NaClO_4_/Fly Ash, is shown in Figure 6. The suitability between the polymer matrix and the fly ash filler has a major impact on the ionic conductivity of CS- and PEO-based electrolyte composites [31]. Figure 6a shows the morphology of pure CS/PEO. The SPE film with the addition of NaClO_4_ showed a smoother surface, Figure 6b. The addition of fly ash caused the surface of the SPE composite to become rougher due to the presence of homogeneously dispersed filler, Figure 6c. The elemental mapping of the SPE composite showed homogeneous distributions of Na and Si, as observed in Figure 6d,e.

The addition of NaClO_4_ and FA increased the ionic conductivity. Pure CS/PEO had a conductivity of 5.61 × 10^−8^ S cm^−1^ and, after the addition of NaClO_4_ salt with a ratio of CS:PEO:NaClO_4_ = 3:2:7.5 (optimal ratio), this increased to 1.02 × 10^−4^ S cm^−1^. The highest ionic conductivity was obtained by the addition of NaClO_4_ at a concentration of 5 wt%: the conductivity value was 3.20 × 10^−4^ S cm^−1^. The ionic conductivity increased because fly ash suppresses the crystallization of the host polymer and creates amorphous regions so that the mobilization of Na^+^ ions is accommodated. The steric hindrance effect was generated when the FA causing the SPE has an amorphous phase, and when Na^+^ ion transport occurs via the interchain and intrachain charge transfer in the amorphous phase [32].

**Figure 7:**
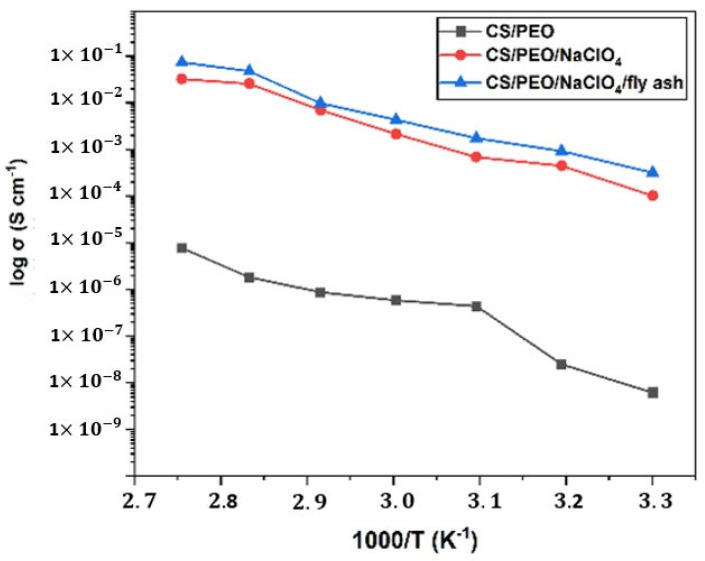
The relationship between temperature and ionic conductivity.

Ionic conductivity increased as the temperature increased. This was because the increase in temperature increased the charge mobilization in the polymer chains in the amorphous phase. Figure 7 shows the relationship between ionic conductivity and the temperature of the SPE. The highest ionic conductivity was achieved from SPE + fly ash at a temperature of 60 °C, which corresponded to 4.34 × 10^−3^ S cm^−1^. The conductivity and temperature relationship of the SPE can be attributed to the data from DSC, where the ionic conductivity decreased at temperatures above the melting point of the SPE.

**Figure 8:**
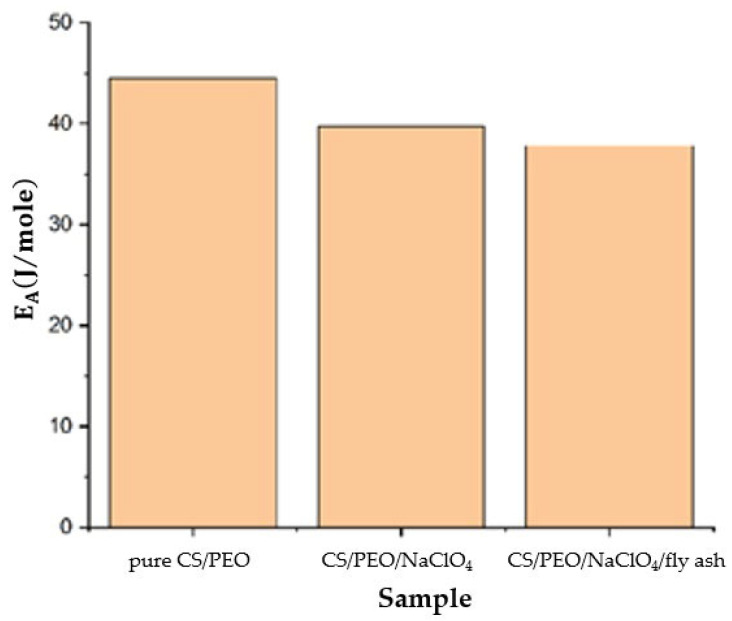
Activation energy for pure CS/PEO, CS/PEO/NaClO_4_ and CS/PEO/NaClO_4_/fly ash.

The activation energy (Ea) can be calculated using the Arrhenius equation as stated in Equation (3):

(3) $$\sigma =\sigma_{0}\exp\;\left(-\frac{Ea}{RT}\;\right)$$

where σ is the ionic conductivity, $\sigma_{0}$ is the pre-exponential factor, Ea is the activation energy, T is the temperature in Kelvin, and R is the ideal gas constant. The Ea calculation results of the SPE are shown in Figure 8. Pure CS/PEO showed the highest activation energy. The smaller Ea was obtained after the addition of NaClO_4_ and fly ash. This indicated that ion mobilization in the SPE requires less energy than pure CS/PEO, so the ionic conductivity was increased [32].

## 4. Conclusions

The solid polymer electrolyte (SPE) composite consisting of CS, PEO, NaClO_4_, and fly ash has been successfully synthesized using the solution casting method. The SPE XRD diffractogram characteristic peaks at 2θ = 11.42°, 18.14°, 19.18°, and 22.96° showed a decrease in crystallinity when NaClO_4_ and fly ash was added. This was confirmed by the DSC melting point at 180.23 °C. FTIR analysis showed that the peak at 600–650 cm^−1^ was the peak of ClO_4_^−^. SEM analysis showed that the surface of the composite becomes rougher upon the addition of fly ash. This indicated a homogeneous dispersion of fly ash. The optimal ionic conductivity was 2.13 × 10^−3^ S cm^−1^ at 60 °C at a ratio of CS:PEO: NaClO_4_ = 3:2:7.5. The addition of the fly ash filler at a concentration of 5% w/w increased the ionic conductivity to 4.34 × 10^−3^ S cm^−1^ at 60 °C.

## Figures and Tables

**Table 1:** Variation of CS/PEO/NaClO_4_ ratio for salt optimization.

Sample Code	CS:PEO:NaClO_4_ Ratio
SPE1	3:2:0
SPE2	3:2:4.5
SPE3	3:2:6
SPE4	3:2:7.5
SPE5	3:2:9
SPE6	3:2:10.5

**Table 2:** Fly ash composition from PT. Paiton.

Compounds	Fly Ash Composition (%)
SiO_2_	33.55
CaO	23.10
Fe_2_O_3_	16.00
Al_2_O_3_	15.94
MgO	4.69
SO_3_	2.24
Na_2_O	1.42
TiO_2_	1.22
K_2_O	0.90
P_2_O_5_	0.36
SrO	0.23
Mn_2_O_3_	0.23
Cl	0.07
ZnO	0.03
Cr_2_O_3_	0.01

**Table 3:** Melting point enthalpy (∆*H_m_*) and crystallinity degree of SPE.

Sample	*T_m_* (°C)	∆*H_m_* (J/g)	*X_c_* (%)
CS/PEO	180.23	31.12	38.3
CS/PEO/NaClO_4_	176.51	29.25	36.02
CS/PEO/NaClO_4_/fly ash	175.53	26.89	31.84

## Data Availability

Not applicable.
